# Awareness and healthcare seeking behavior of neonatal danger signs, and predictor variables among mothers/caregivers in four developing regional state of Ethiopia

**DOI:** 10.1186/s12887-024-04656-8

**Published:** 2024-03-16

**Authors:** Nagasa Dida, Lonsako Abute, Tariku Dejene, Tolasa Yadate, Temesgen Geleta, Rachana Sharma, Hnin Su Mon, Tesfaye Simireta, Hailemariam Addisu

**Affiliations:** 1 Department of Public Health, Medicine and Health Science College, Ambo University, P.O.B: 19, Ambo, Ethiopia; 2 School of Public Health, Medicine and Health Science College, Wachemo University, Hossana, Ethiopia; 3 Center for Population Studies, College of Development Studies, Addis Ababa University, Addis Ababa, Ethiopia; 4 Department of Public Health, Medicine and Health Science College, Dilla University, Dilla, Ethiopia; 5 School of Public Health, St. Paul’s Hospital Millennium Medical College, Addis Ababa, Ethiopia; 6 Social and Behavioral Change Program, UNICEF, Addis Ababa, Ethiopia; 7 Health Education and Promotion Team, Federal Ministry of Health, Addis Ababa, Ethiopia

**Keywords:** Awareness, Healthcare seeking behavior, Neonatal danger signs, Developing regional state, Ethiopia

## Abstract

**Introduction:**

: Mothers/caregivers should be aware of a newborn’s danger signs and promptly seek medical attention. Hence, this study assessed mothers’/caregivers’ awareness, healthcare seeking behaviors for neonatal danger signs and their determinants in the developing regional state of Ethiopia.

**Methods:**

A community-based cross-sectional study was employed among mothers/caregivers of neonates in the developing regional state of Ethiopia. The sample was determined in collaboration with the Central Statistics Agency of Ethiopia. Stratified multi-stage cluster sampling was used to recruit the sample. Data were collected through an interviewer administered structured questionnaire using a tablet computer. Descriptive statistics and binary logistic regression were applied to identify determinants of awareness and treatment-seeking behavior for neonatal danger signs.

**Results:**

The result of the study showed that nearly one-third (32.7%) of the respondents had a good level of awareness of neonatal danger signs, and 69.0% of the respondents had good healthcare-seeking practice about neonatal danger signs. Regional state (Benishangul-Gumuz) [AOR = 1.61; 95% CI (1.09, 2.39)], Muslim’s [AOR = 1.75; 95% CI (1.20, 2.55)] and permission to travel to a health facility [AOR = 0.48; 95% CI (0.37, 0.63)] were determinants of mothers’/caregivers’ awareness about neonatal danger signs. Antenatal care (ANC) attendance and institutional delivery were shown to have a positive association with neonatal healthcare seeking (AOR = 2.14 and AOR = 2.37, respectively).

**Conclusion:**

In Ethiopia’s developing regional states, mothers/caregivers were remarkably unaware of neonatal danger signs. Region, religion, mothers’/caregivers’ age, and need for permission to travel to a health facility were predictor variables for neonatal danger sign awareness. Better healthcare seeking practices, which are determined by ANC attendance and institutional delivery, are observed in these regions. Federal and regional governments should give these regions due attention. Moreover, regional health bureaus and health professionals should tackle the problem by focusing on the identified factors.

## Introduction

The first 28 days after birth are the most critical time of human life. Annually, 2.6 million infants die before reaching 1 month across the globe [1]. On this basis, the World Health Organization considers efforts to address inequities as key components of global efforts aimed at improving newborn and child health and survival rates [2]. According to the United Nations Inter-Agency Group for Child Mortality Estimation 2020 report, the death proportion of neonates globally on average is 17 deaths per 1,000 live births in 2019, and it has dropped by 52% from 38 deaths per 1,000 in 1990 [3]. In addition, the report reveals that over 6,700 children pass away every day and that almost 2.4 million children die in their first month of existence [4]. About 75% of newborn deaths in 2019 happened within the first week following birth, and 33.33% happened within the first day[5].

In industrialized countries, there are less than eight child deaths per 1,000 live births, compared to 76 per 1,000 in sub-Saharan Africa [6, 7]. Research demonstrates that women’s decision-making autonomy is low, particularly in developing nations. Yet, increasing women’s influence in decision-making results in better healthcare access, poverty reduction, and household economic growth. In fact, women are largely viewed as the primary caregivers in low-income nations [8–10]. Although most neonatal deaths occur at home, few families seek medical attention for symptoms of neonatal sickness, and almost no neonates are brought to medical facilities when they are ill. Delayed healthcare seeking significantly increases neonatal mortality [11].

Healthcare services for mothers and children are a crucial determinant of their health outcomes [12]. Recent initiatives in Ethiopia to enhance newborn health outcomes have had positive effects. Newborn and infant mortality have steadily decreased as a result of other strategic efforts and the country’s health sector transformation plan being implemented. Ethiopia has decreased neonatal mortality from 39 to 30 deaths per 1,000 live births and infant mortality from 77 to 43 per 1,000 live births between 2005 and 2019. The most critical period for a child’s survival is, most importantly, the first 28 days of life [13]. Notwithstanding this outstanding accomplishment, Ethiopia still has 191,000 under-five fatalities annually [14]. Pneumonia (17%), diarrhea (8%), and newborn causes (47%) account for the majority of these fatalities, with malnutrition serving as a significant underlying risk [15].

Mothers´ and husbands´ higher educational achievement, living close to health facilities, previous experience of neonatal danger signs, ANC and PNC attendance, and access to television for information were positively associated with mothers´ good knowledge about neonatal danger signs [16, 17].

Caretakers in Ethiopia failed to seek medical attention when neonatal danger signs were present, which led to an increase in neonatal deaths at home [11]. The socioeconomic, emotional, fertility decisions, contraceptive usage, and sexual lifespan of women are impacted by poor participation in decision-making processes and limited access to health services, which leads to a demographic problem [18]. Utilization rates for health care services can be influenced by knowledge of disease origins, symptoms, and treatments, trust in providers. These factors can impact neonatal health. Lack of knowledge, perceived low-quality medical care, and parents’ inadequate education may cause parents to misjudge the effectiveness of care and lose faith in the medical system, which may drive them to turn to traditional healers [19, 20]. . Access to health services, such as availability of services, geographic accessibility, travel time, and affordability determine child health inequity. These factors mostly favour those who live closer to health facilities, and those who can afford both the direct and indirect costs of the service as well as the opportunity costs associated with receiving the service [21].

Identifying early maternal health care-seeking habits and understanding the characteristics associated with health care-seeking behavior for neonatal danger signals is crucial for nations such as Ethiopia, especially developing regional states. However, compared to the national average, developing regional states in Ethiopia (Afar, Benishangul-Gumuz, Gambella, and Somali) have demonstrated less improvement in critical health outcomes. Health activities, particularly health extension projects, were limited by distance, geography, topography, weather conditions, and security concerns in some regions and were not tailored to the unique settings of pastoralist communities. In Ethiopia’s developing regional states, there is still a great deal of work to be done to reduce morbidity and mortality rates, encourage newborn health care seeking, and foster a supportive attitude towards the diagnosis, treatment, and prevention of neonatal disorders. Thus, the goal of this study is to evaluate the factors that influence newborn healthcare seeking behavior in four of Ethiopia’s growing regional states.

## Methods

### Study setting and design

The study was conducted in the four developing regional states of Ethiopia, namely Gambela, Afar, Benshagu-Gumuz, and Somali. Regions with less developed infrastructure, sluggish growth in many sectors, and border disputes further complicate development. A community-based cross-sectional study design was conducted among 6,706 mothers and primary caregivers of children aged below five years in February 2021.

The study participants included mother-child pairs or primary caregivers of children aged 15 years and older.

### Sample and sampling techniques

The sample was determined in collaboration with the Central Statistics Agency (CSA) of Ethiopia. To get an adequate sample, the necessary statistical power for analysis was considered, including a sub-sample of refugees living in camps or refugee settlements in each Developing Regional State (DRS). The assumptions used to determine the sample were: women’s awareness of neonatal danger to be 40.7% in Ethiopia [22], a 95% confidence level, a design effect of 3, a 5% non-response rate, which yielded a total sample size of 3246.

A stratified multi-stage cluster sampling design was applied with a target sample size from which 3,104 mother**/**caregivers fully responded to the survey and were aged 15 years and over. To recruit the study subjects, the sample was drawn by simple random sampling from the sampling frame of enumeration areas (EAs) prepared by the CSA in 2018–2019 for the upcoming Ethiopian population census. For the refugee sample, the CSA performed a simple random selection of camps/settlements from those located in each DRS. Unlike the general population, the CSA could not provide a frame for refugee households. As a result, 600 refugee samples were selected through a random route approach with a fixed selection interval of 5 households. Similar proportions of samples were recruited in each refugee camp in each developing regional state.

### Data collection method and tool

The data were collected using a structured questionnaire administered by a professional interviewer using a tablet. Prior to the start of fieldwork, six days of training, including two days of field practice with the questionnaire were given to the data collectors. To identify the variable predictors of child healthcare service utilization, a socio-ecological model was used. Healthcare service variables like postnatal care, early healthcare seeking for neonatal danger signs, vaccination, knowledge, and response to common childhood illness danger were accordingly assessed for the sub-group targets (0–11 months, 12–23 months, and 24–59 months).

### Operational definition

Symptoms that indicate a newborn baby should be taken to a health facility immediately were used to measure mothers’/caregivers’ awareness of neonatal danger signs; health seeking behavior of the mothers and caregivers was assessed whether they sought treatment for their baby when they faced the neonatal danger signs.

### Data quality management and analysis

Data were entered in the field using Open Data Kit (ODK) data collection software while collecting the data, and they were exported to Excel then exported to SPSS version 26 for analysis. Data were cleaned, and descriptive statistics like frequencies, percentages, and proportion were done for the categorical variables. The mean and standard deviation were calculated for the continuous variable. The final model was developed using bivariate logistic regression at a p-value of 0.2. These variables were fitted to multivariable logistic regression to identify predictor variables for child healthcare service utilization. A P-value of 0.05 was used to declare an association between the explanatory and dependent variables as a cutoff point. For each sub-group of the study participants, independent analysis was done.

Because of the unavailability of a sampling frame for refugees, the refugee sample is unweighted. Refugee statistics are not included in the weighted total for the DRS. Consequently, the refugee statistics are shown in a column separated from the main, and only aggregated statistics are presented for the refugee population because the sample of refugees is too small for regional disaggregation.

## Result

### Awareness about neonatal danger signs

From the total mothers/caregivers of neonates included in the study, nearly three-thirds of them were in the age range of 25–34 years. 78% and 60% of the mothers attended antenatal care and gave birth in a health facility, respectively (Table 1).

**Table 1:** Socio-demographic variables of mothers/care givers of neonates in developing regions of Ethiopia, February, 2021

Socio-demographic variables	Awareness about neonatal danger signs		
**Regional state**	No	Yes	Total
Afar	195	112	307
Benishangul-Gumuz	203	104	307
Ethiopia Somali	197	101	298
Gambela	230	84	314
Refugee	150	48	198
**Age of the Mother /caregivers**			
<25	361	151	512
25–34	374	205	579
35+	90	45	135
**Religion**			
Christian	310	117	427
Muslim	472	273	745
Others	43	11	54

Based on 1,226 births that were delivered in the four developing regional states of Ethiopia in the year before the survey, the analysis was conducted. According to the analysis’s findings, nearly a third (32.7%) of respondents knew a good deal about neonatal warning signs. Of the refugees, 24% have good awareness about neonatal danger signs. Those participants within 25–34 years of age groups were with high proportion of awareness towards neonatal danger signs. When we see trust and frequency of information received towards neonatal danger signs, mass media and print communication materials (TV, Radio, Newspaper, SMS, Leaflet, and Poster) had a mean score of 7.63, the health workforce had a mean score of 19.50, the health institution had a mean score of 7.31, and other sources, including the community, had a mean score of 21.61. In this study, there is a 0.327 proportion with good awareness about danger signs. There are 0.061 Pseudo r-squared and Prob > chi2 is 0.000, 94.071 Chi-square and 1483.817 Akaike crit. (AIC) and Bayesian crit. (BIC) of 1555.379.

In the multivariable analysis, respondents from Benishangul Gumuz had an increased likelihood of having a good level of awareness regarding neonatal danger signs [AOR = 1.61 with 95% CI (1.09, 2.39)]. Similarly, refugees were significantly less likely to have good awareness of neonatal danger signs than non-refugees [AOR = 0.62 with a 95% CI (0.43, 0.90)]. Muslim respondents, compared to Christians, had a 75% increased likelihood of having a good awareness of neonatal danger signs [AOR = 1.75 with a 95% CI (1.20, 2.55)]. Women who required permission to travel to health facilities were significantly less likely to have a good understanding of neonatal warning signs compared with women with flexibility [AOR = 0.484, 95% CI 0.37, 0.63]. The level of trust and frequency of health information received from health institutions and the community promotes the level of awareness about neonatal danger signs. An increase in the score of trust and frequency of health information from health institutions positively increased the likelihood of awareness about neonatal danger signs (AOR = 1.01 with a p-value < 5%). Moreover, an increase in the score of trust and frequency of health information received from community sources improved the chance of having a good level of awareness about neonatal danger signs (AOR = 1.02 with a p-value < 5%) (Table 2).

**Table 2:** Multivariable logistic regression analysis result for awareness about neonatal danger signs among mothers/caregivers of neonate in developing regional states of Ethiopia, February, 2021

Characteristics and categories	Awareness about neonatal danger signs		COR	AOR	95% CI		Sig
	No (*n* = 825)	Yes (*n* = 401)			LL	UL	
Region *[Afar = Ref]*	195	112	1	1			
Benishangul Gumuz	203	104	0.89	1.61	1.09	2.39	0.017**
Ethiopia Somali	197	101	0.89	1.20	.83	1.74	0.342
Gambela	230	84	0.64^***^	1.36	.85	2.183	0.199
Refugee	150	48	0.61^***^	0.62	.43	.90	0.011**
Age of the Mother [*< 25 = Ref*]	361	151	1	1			
25–34	374	205	1.31^**^	1.258	.96	1.64	0.091*
35+	90	45	1.20	1.10	.72	1.68	.664
Religion [Christian = Ref]	310	117	1	1			
Muslim	472	273	1.53^***^	1.75	1.2	2.55	0.004***
Others	43	11	0.68	.71	.35	1.46	.352
Asks permission to travel to health facility [No = Ref]	430	261	1	1			
Yes	395	140	0.58^***^	0.48	.37	.63	0.000***
Trust and Frequency of Information Receipt	***Mean (SE)***	***Mean (SE)***					
Media (TV, Radio, Newspaper, SMS, Leaflet, and Poster)	6.74 (0.31)	7.63 (0.47)	1.01	.10	.98	1.01	.583
Health Workforce	16.88 (0.41)	19.50 (0.55)	1.020^***^	1.01	1.00	1.02	.272
Health Institution	4.75 (0.29)	7.31 (0.64)	1.024^***^	1.01	1	1.03	0.044**
Other sources including community	16.00 (0.54)	21.61 (0.84)	1.021^***^	1.02	1.01	1.03	0.000***
Proportion with good awareness about danger signs		0.327	Number of observations			1226	
Pseudo r-squared		0.061	Prob > chi2			0.000	
Chi-square		94.071					
Akaike crit. (AIC)		1483.817	Bayesian crit. (BIC)			1555.379	

*** *p* < .01, ** *p* < .05, * *p* < .1

### Neonatal health care seeking

Regarding neonatal healthcare seeking, the analysis was based on 403 neonates that showed danger signs in the last four weeks after birth. Among these, 69.0% of the respondents had good neonatal healthcare seeking practices. Greater than 78% of the participants who gave birth at a health institution have good neonatal healthcare-seeking practice while only 54.3% of the participants who did not give birth at a health institution have good neonatal healthcare seeking practice. Regarding the proportion of neonatal health care seeking behavior by region, Gambela was the highest at 30.57% and Afar was the lowest at 16.18. In this study, 0.690 proportion of people who are aware of danger signs, there is a 0.090 pseudo-r-squared, a probability of > chi2 of 0.000, a 45.080 Chi-square, an Akaike critical of 470.027. (AIC), and a Bayesian crit. (BIC) of 502.019.

The results from the multivariable logistic regression showed that the practice of neonatal health care seeking was highly prevalent in Gambela (AOR = 2.26 with a p-value < 5%). ANC attendance and institutional delivery were shown to have a positive association with neonatal healthcare seeking, AOR = 2.14 and AOR = 2.37, respectively (Table 3).

**Table 3:** Multivariable logistic regression analysis result for neonatal health care seeking practice of mothers/care givers of neonates in developing regions of Ethiopia, February, 2021

Characteristics and categories	Neonatal Health Care Seeking		COR	AOR	95% CI		Sig
	No (*n* = 125)	Yes (*n* = 278)			LL	UL	
Region *[Afar = Ref] 16.18*	26	45	1	1			
Benishangul Gumuz 24.10	34	67	1.14	1.01	.52	1.97	0.971
Ethiopia Somali 29.13	48	81	0.98	1.30	.68	2.47	0.426
Gambela 30.57	17	85	2.889^***^	2.26	1.08	4.73	0.031^**^
Age of the Mother [*< 25 = Ref*]	55	121	1	1			
25–34	50	132	1.200	1.40	.86	2.27	0.178
35+	20	25	0.57^*^	0.78	.38	1.61	0.503
ANC [*No = Ref*]	45	45	1	1			
Yes	80	233	2.91^***^	2.14	1.25	3.66	0.0105^***^
Institutional Delivery [*No = Ref*]	74	88	1	1			
Yes 78.8%	51	190	3.13^***^	2.37	1.46	3.85	0.001^***^
Proportion with good practice		0.690	Number of observations			403	
Pseudo r-squared		0.090	Prob > chi2			0.000	
Chi-square		45.080					
Akaike crit. (AIC)		470.027	Bayesian crit. (BIC)			502.019	

*** *p* < .01, ** *p* < .05, * *p* < .1

## Discussion

In the rapidly emerging regional state of Ethiopia, this research sought to assess mothers’ and primary caregivers’ awareness of neonatal danger signs and healthcare seeking behavior for neonatal sickness. The study confirmed that a third (32.7%) of mothers/caregivers had good awareness of neonatal danger signs. This study is in line with the study findings from Madagascar, where 31.2% of themothers had good knowledge of neonatal danger signs [20]. This result is significantly lower than that of a study conducted in Kigali, Rwanda, health centers, where 67% of parents knew something about neonatal danger signs [23]. Sociocultural differences and the study setting could be the reasons for the variance. Similar research from a remote Pakistani community revealed that 76% of child care providers were aware of the symptoms of diarrhea. On the other hand, just 21% of the same caregivers were aware that pneumonia might cause breathing difficulties [24]. Age difference for the concerned children, and awareness of childhood illnesses’ symptoms could be a justification for the difference. This is also lower than a study from a rural county in the southwest of China (42%) [25]. The level of development in the country could be the reason for the difference.

Furthermore, many studies from other parts of Ethiopia — 40.8% in Dire Dawa [26], 39% in Sheko District in Southwest Ethiopia [27], 48.1% in Gidan District Health Centers, North Wollo, Ethiopia [28], 40.7% in Gurage Zone, Southern Ethiopia [29] and 50.2% in Southern Ethiopia [30] —found that mothers or caregivers were more aware of neonatal danger signs. These investigations were conducted in Ethiopia’s developed regional states, where improved health information access is anticipated. Sociocultural distinctions between Ethiopia’s developed and emerging regional states may be largely responsible for the noticeable discrepancies.

Among the main sources of information for mothers and other people who care for children were health professionals, media and communication materials such as (TV, Radio, Newspaper, SMS, Leaflet, and Poster), and other sources such as community platforms. This study is supported by research done in health centers in Kigali, Rwanda, where neighbors, media outlets like TV, radio, and magazines, as well as healthcare professionals and community health extension workers, are primary sources of information for parents of children about neonatal danger signs [23]. Studies conducted in other regions of Ethiopia revealed the same results [26, 30, 31].

Mothers’ or caregivers’ awareness of neonatal danger signs is significantly influenced by factors such as region, being a refugee, religion of the respondents, permission required to travel to a health facility, and mothers’ or caregivers’ trust in and use of information from health professionals. Respondents from refugee camps, Christian religion followers, and respondents who needed permission to travel to a health facility were less likely to have good neonatal danger sign awareness. Responses from refugee camps and those who need permission to visit healthcare facilities are likely to have limited access to information. Similar to this study finding, ethnic groups in China [25] and information from healthcare facilities in Kigali, Rwanda [23] were among the factors influencing neonatal danger signs in mother or child care providers. Unfair information service dissemination among the region or ethnic group and cultural differences in information seeking among the region or ethnic group could be justified. A study from Northern Wollo Ethiopia and Southeastern Ethiopia found that health education and counseling can significantly increase awareness of neonatal danger signs among mothers and child care caregivers [28, 30].

According to the study, 69.0% of mothers and caregivers in Ethiopia’s emerging regional states sought neonatal healthcare. This result is significantly higher than the treatment-seeking behavior of mothers and other caregivers of sick children in three separate studies from Nigeria, which were 31.4%, 36%, and 18.6% [27, 32, 33]. The three studies from Nigeria examined specific pediatric diseases, and the older age of the children in those studies may have contributed to the difference. Additionally, the context of the study and cultural differences may also be factors. The appropriate healthcare seeking behavior for childhood illness among mothers in Malawi (61%) and Northern Uganda (50%) was significantly lower than this study’s findings [34, 35]. Similarly, studies on mothers or caregivers from Northwest Ethiopia [36], Woldia Town Administration, Northeast Ethiopia [30], and Efratana Gidim District, East Amhara, Ethiopia [37] had lower appropriate healthcare seeking behavior for common childhood illnesses. Experiencing older children’s illness could also delay mothers or caregivers from good or appropriate healthcare seeking.

The history of antenatal care follow-up, institutional delivery, and region of the respondents were the predictor variables for neonatal healthcare seeking. However, in most of the similar studies, these variables were not among those have an association with healthcare seeking for neonatal illness except the region, which was also a predictor variable in a study conducted in Malawi [35]. Similar to this study, the age of mothers or caregivers was not statistically associated with healthcare seeking behavior [31, 38, 39].

## Conclusion and recommendation

Awareness of mothers/caregivers about neonatal danger signs was remarkably lacking in developing regional states (Gambella, Benishangul-Gumuz, Afar, Somali and refugees from these regions) of Ethiopia. Mothers in the Gambella region and refugee camps have lower neonatal danger signs awareness. Region, religion, and the request for permission to travel to a health facility continue to be the best predictor of neonatal danger sign awareness. Despite low awareness of neonatal danger signs among mothers and caregivers, better healthcare seeking practices are observed in these regions. Contrary to mothers’ awareness of neonatal danger signs, healthcare seeking practice is encouraged in Gambella. Despite the fact that better healthcare seeking is expected among mothers with better awareness of neonatal danger signs, a large proportion was observed among mothers with low awareness. The history of antenatal care follow-up, institutional delivery utilization and the region have significant roles in healthcare seeking for neonatal danger signs.

The federal government, regional state administrations, and stakeholders in these regions should give due consideration to these regions. Health extension workers and different health professionals are expected to provide information on neonatal danger signs for mothers/caregivers at any opportunity. This is to increase awareness of neonatal danger signs for better healthcare seeking practices. The identified factors should be taken into consideration at all stages of the intervention. It is recommended that researchers conduct a further study with a strong design.

## Data Availability

The datasets generated during and/or analyzed during the current study are available from the corresponding author on reasonable request.

## Abbreviations

ANC: Antenatal Care
AOR: Adjusted Odd Ration
CI: Confidence Interval
COR: Crude Odd Ratio
CSA: Central Statistics Agency
DRS: Developing Regional States
LL: Lower Limit
Ref.: Reference group
SPSS: Statistical Package for the Social Sciences
TV: Television
UL: Upper Limit
UNICEF: United Nations International Children’s Emergency Fund
